# Metabolic Syndrome and Its Characteristics among Reproductive-Aged Women with Polycystic Ovary Syndrome: A Cross-sectional Study in Northwest Iran

**Published:** 2013-03-03

**Authors:** Fatemeh Pourteymour Fard Tabrizi, Beitollah Alipoor, Mahzad Mehrzad Sadaghiani, Alireza Ostadrahimi, Aida Malek Mahdavi

**Affiliations:** 1 Nutrition Research Center, Tabriz University of Medical Sciences, Tabriz, Iran; 2 School of Nutrition, Tabriz University of Medical Sciences, Tabriz, Iran; 3 Alzahra Hospital, Infertility and IVF Center, Tabriz University of Medical Sciences, Tabriz, Iran

**Keywords:** Metabolic Syndrome, ATP III Criteria, Polycystic Ovary Syndrome

## Abstract

**Background:**

Metabolic syndrome (MetS) is a clustering of factors known to increase the risk for cardiovascular disease (CVD) and diabetes mellitus. Polycystic ovary syndrome (PCOS), the most common endocrine disorder among reproductive-aged women, is also closely linked to MetS. Limited information is available pertaining to the prevalence of MetS in Iranian PCOS women; therefore this study assesses the frequency of MetS and its components among PCOS women from Tabriz, Iran.

**Materials and Methods:**

In this cross-sectional study, we evaluated a total of 200 women with PCOS who referred to the only specialty and subspecialty gynecological center in Northwestern Iran. PCOS was diagnosed according to Rotterdam criteria. This study defined clinical and biochemical parameters for MetS by the National Cholesterol Education Program Adult Treatment Panel III (NCEP ATP III) criteria. Statistical analyses were performed with descriptive-analytical methods using SPSS software version 16.

**Results:**

MetS was identified in 39.5% of PCOS women. The frequencies of individual components of MetS among studied subjects were: high-density lipoprotein cholesterol level (HDL-C)<50 mg/ dL (99.5%), waist circumference(WC) ≥88cm (65%), triglycerides (TG) ≥150 mg/dL(98%), and blood pressure≥130/85 mmHg(34%).There were no fasting glucose concentrations≥110 mg/dL. The frequency of MetS increased with body mass index (BMI)as follows: normal (5.4%), overweight (41.5%) and obese (85.7%) women (p<0.0001).

**Conclusion:**

The PCOS women in this study had a high frequency of MetS and its individual components, particularly decreased HDL-C and increased triglyceride levels. These data can useful for lifestyle modification programs.

## Introduction

Polycystic ovary syndrome (PCOS) as the most common endocrinopathy among reproductiveaged women is a major health and economic burden (1). Depending on the criteria used for its definition, the method used to define each criterion and the study population, the prevalence of PCOS ranges between 2.2 to 26% in various countries (2). Prevalence of PCOS among Iranian women of reproductive age has been determined to be 15.2% according to Rotterdam Criteria (3).

This ovarian dysfunction syndrome encompasses a broad spectrum of clinical signs and symptoms. Clinical manifestations include menstrual irregularities, hyperandrogenism and infertility (4). According to previous reports, insulin resistance, obesity and dyslipidemia have commonly been described as associated with PCOS (5). These disorders are also the features of the so-called metabolic syndrome (MetS) or syndrome X, another cluster of endocrine disturbances defined by the World Health Organization, the National Cholesterol Education Program Adult Treatment Panel III (NCEP ATP III) and the International Diabetes Federation (IDF) guidelines (6).

MetS is a combination of cardiovascular risk factors, including dyslipidemia, impaired fasting glucose levels, abdominal obesity and high blood pressure. Insulin resistance, as a major defect in MetS, appears to be a common linkage between these coexisting abnormalities (7). Since the anthropometric and metabolic abnormalities found in PCOS overlap with the components of MetS (8,9), the issue regarding MetS in women with PCOS has generated tremendous interest. Diagnosis of MetS requires clinical and laboratory information that are grouped into criteria. However, each institute defines the cut-off for each criterion differently. Such difference would affect the prevalence of MetS, even within the same population (10). Recent studies have found a much higher prevalence of MetS in women with PCOS than in those without PCOS (8,11,12). According to estimates based on the US population, the prevalence of MetS in women with PCOS is approximately 43 to 46% (13-15). Within this context, limited data are available regard the prevalence of MetS and its components in Iranian women with PCOS, particularly in Tabriz, a city in Northwestern Iran. The present study aims to determine the frequency of MetS and its individual components according to NCEP ATPIII criteria (16) among reproductive-aged PCOS women in Tabriz, Northwestern Iran.

### Materials and Methods

#### Study design and subjects

This cross-sectional study was conducted on 200 women aged 20-40 years old who were diagnosed with PCOS by a gynecologist, according to Rotterdam criteria (17) with the presence of at least two of the three following features: polycystic ovaries, oligo- or anovulation (characterized by oligomenorrhea or amenorrhea), hyperandrogenism (clinical and/ or biochemical features) and the exclusion of other disorders such as nonclassical congenital adrenal hyperplasia, thyroid dysfunction, and hyperprolactinemia. Other exclusionary criteria were unresolved medical conditions such as renal or hepatic dysfunction. The use of medications known or suspected to affect reproductive or metabolic function such as hormonal medications, statins, thiazolidinediones, corticosteroids, anti-obesity drugs, metformin, vitamin and mineral supplements within 60 days of study entry was prohibited. Volunteers were selected at Al-Zahra University Hospital from 2008 to 2010. All subjects in the study period were evaluated in the study census. Patients were referred to this hospital for treatment for infertility and irregularity of menses. Al-Zahra University Hospital, located in Tabriz, is the only specialty and subspecialty Gynecological Center in Northwestern Iran. This facility provides secondary and tertiary care for patients. This study was approved by the Institutional Review Board and Ethical Committee of Tabriz University of Medical Sciences, Iran (5/4/2484) and written informed consent was obtained from the subjects.

MetS was defined using the definition of the NCEP ATP III (16) with the presence of three or more of the following abnormalities: waist circumference(WC)≥88 cm; fasting serum glucose of at least 110 mg/dL; fasting serum triglycerides (TG)≥150 mg/dL; serum high-density lipoprotein cholesterol (HDL-C)<50 mg/dL, and blood pressure≥130/85 mm Hg. All subjects underwent a clinical examination where body weight, height, waist and hip circumferences,and blood pressure were measured.

#### Anthropometric measurements and blood pressure

Body weight was measured to the nearest 0.1 kg using a calibrated Seca Scale (SECA 707; HH, Modena, Italy), with the participants barefoot and wearing light clothing. Standing height was measured to the nearest 0.1 cm using a mounted tape. The participants were barefoot with arms hanging freely at their sides. Body mass index (BMI) was calculated as weight in kilograms divided by the square of height in meters (kg/m^2^). According to the World Health Organization categories, persons who have a BMI between 25.0 and 29.9 are classified as overweight and those who have a BMI of 30.0 or higher are classified as obese (18). Waist circumference was measured at the narrowest level over light clothing with a precision of 0.1 cm using an unstretched tape measure, without any pressure to body surface. Hip circumference was determined as the maximum value over the buttocks. In order to calculate the waist/hip ratio, we divided the WC by the hip circumference.

Systolic and diastolic blood pressures (SBP and DBP) were measured twice in the right arm in a sitting position after a 10 minute rest period, using a mercury sphygmomanometer the average of the two measurements was used for analysis.

#### Laboratory measurements

Blood samples were collected after a 12-hour overnight fast. Serum glucose was measured by an enzymatic colorimetric method. Serum total cholesterol and TG were determined by using commercially available enzymatic reagents (Pars Azmoon, Tehran, Iran) adapted to an autoanalyzer (model Alcyon 300 Abbott, USA and Germany). High-density lipoprotein cholesterol was determined after precipitation of the apolipoprotein B–containing lipoproteins with phosphotungstic acids. Low-density lipoprotein cholesterol (LDL-C) was indirectly measured by using the Friedewald formula (LDL=total cholesterol - HDL - TG/5).

#### Statistical analysis

All data were collected in a cross-sectional survey. Continuous variables were presented as mean and standard deviation (SD), while categorical variables were presented as frequency (number) and percentage. Trend chi-square analysis was used for comparison of categorical variables and percentages of study variables among BMI and age categories. P<0.05 was considered significant. Statistical analysis was performed with SPSS software version 16.0.

### Results

In this study patients’ mean ± SD for age was 26.18 ± 4.27 years, BMI was 27.12 ± 2.34kg/m^2^ and WC was 91.08 ± 8.5cm. The overall prevalence for overweight was 71% (142 out of 200) whereas obese patients comprised 10.5% (21 out of 200)of the study population. The frequency of MetS as determined by NCEP ATP III was 39.5% (79/200). A considerable proportion (96.7%) of women without MetS (n=121) fulfilled two criteria whereas 3.3% (4/121) fulfilled one criteria for MetS. Approximately 70% of patients with MetS had three criteria and 30% had four MetS criteria.

**Table 1:** Age-stratified frequency of metabolic syndrome (MetS) in women with PCOS


				

**MetS N (%)**	10 (12.6)	30 (49.1)	39 (65.0)	79 (39.5)
**Non-MetS N (%)**	69 (87.4)	31(50.8)	21(35.0)	121 (60.5)


Frequency of MetS differed across age groups (trend Chi-square test; p=0.03).

According to table 1, the frequency of MetS increased with age, from 12.6% in women aged 20-26 years to 65.0% in those aged 34-40 years (p=0.03). The frequency of MetS was also significantly associated with BMI (p<0.0001; Table 2).

**Fig 1:**
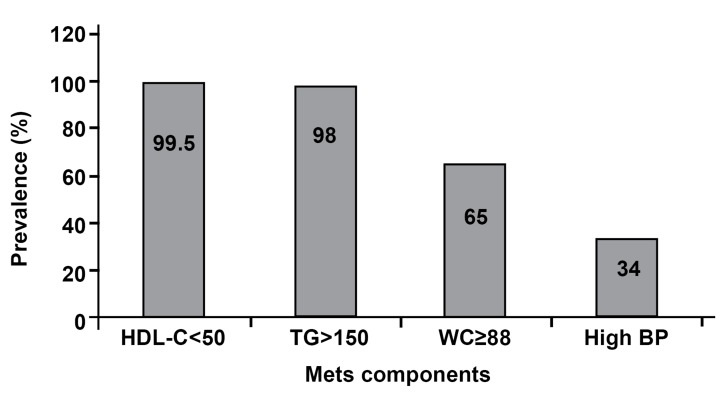
Percentage of metabolic syndrome (MetS) components according to NCEP ATP III criteria in women with PCOS

Compared with the normal BMI group, overweight and obese women had a 7.7-fold and 16- fold increased risk for MetS, respectively. High WC and high blood pressure were also more prevalent in the overweight and obese groups (p<0.0001). The most prevalent isolated abnormality of MetS in women with PCOS was a HDL-C level below 50 mg/dLwhich was observed in 99.5% (199 out of 200) subjects. Other abnormalities included increased serum TG in 98% (196 out of 200), increased WC in 65% (130 out of 200) and hypertension in 34% (68 out of 200). There were no elevated fasting glucose concentrations observed (Fig1).

**Table 2:** Body mass index group-stratified frequency of metabolic syndrome and its components in women with PCOS


	BMI (kg/m^2^)			
	<25 (n=37)	25-29.9 (n=142)	≥30 (n=21)	P valuea

**MetS**	2 (5.4%)	59 (41.5%)	18 (85.7%)	<0.0001
**High blood pressure**	0 (0%)	48 (33.8%)	20 (95.2%)	<0.0001
**Hypertriglyceridemia**	33 (89.2%)	142 (100%)	21 (100%)	0.15
**Low HDL-C**	36 (97.3%)	142 (100%)	21 (100%)	0.19
**Elevated waist circumference**	8 (21.6%)	101 (71.1%)	21 (100%)	<0.0001
**Elevated fasting glucose**	0 (0%)	0 (0%)	0 (0%)	


HDL-C; High-density lipoprotein cholesterol and a; Trend Chi-square test.

### Discussion

Today, many researches are focused on metabolic complications in the field of PCOS, among women. An economic assessment has reported that 40% of the economic costs of PCOS can be attributed to type 2 diabetes mellitus in the USA (19). This emphasizes the necessity for prevention of long-term complications through proper screening, diagnosis and intervention. In this regard, we have conducted this study to determine the frequency of MetS in reproductive-aged women with PCOS according to the NCEPATP III definition (16) among PCOS women from Tabriz, Iran. The frequency of MetS in these women was 39.5%. Delavari et al. conducted a national survey in both urban and rural areas of all 30 provinces in Iran. Their study has shown a prevalence rate of 37.4% for MetS in this population with a higher frequency in women than men (20). However, in our study the prevalence of MetS in a small number of PCOS patients approximated the results (39.5%) of this study that was conducted on a large, general population. Several studies have assessed the prevalence of MetS in women with PCOS. Data regarding the prevalence of MetS in Iranian women with PCOS are limited, and the results vary in studies of different populations. Data from a case-control study conducted by Hosseinpanah et al. has shown that MetS was not frequent in a sample of PCOS Iranian population compared to healthy controls (21). Research by Mehrabian et al. showed the overall prevalence of MetS in different phenotypic subgroups of Iranian PCOS subject according to Rotterdam Criteria was 24.9% (22). The prevalence of MetS in a certain population varies according to the definition of diagnostic criteria. According to the Glueck et al. (13) and Apridonidze et al.(14) studies, the prevalence of MetS in American women with PCOS was 46% and 43%, respectively, both of which were much higher than our study. Conversely, Vrbíková et al. (23) did not find a higher prevalence of MetS in Czech women with PCOS even those women had higher BMI,WC, and blood pressure, and lower HDL-cholesterol levels than women without PCOS. In a study of Italian women, Carmina et al. (24) observed that MetS was more frequent in women with PCOS than in the general population, however this prevalence was much lower than in the United States. According to their study findings, this lower prevalence of MetS intheir study subjects compared to PCOS women from the US suggested that genetic factors, differences in lifestyle and diet pattern influence the prevalence of MetS in women with PCOS profoundly (24).

In this study, the occurrence of low HDL-C was the most frequent component of MetS in women with PCOS, followed by increased serum TG and increased WC. Similar results have been reported in other studies. It has been mentioned that dyslipidemia is the most common metabolic abnormality in PCOS, with a prevalence as high as 70% according to NCEP criteria (14, 25, 26). Importantly, the majority of our subjects (99.5%) had at least one MetS abnormality, a finding similar to the results of Apridonidze et al. (14). In a recent systematic review of 2192 studies by Moran et al., the frequency of the different components of MetS in women with PCOS was elevated WC or BMI (11- 98%), decreased HDL-C (28.6-95%), increased TG (5.5-56%), elevated blood pressure (7.3-70%) and elevated fasting glucose (0-43.5%) (27). These results supported our study findings on the frequency of the MetS components in PCOS women although our study subjects had higher elevated TG compared to the range of this report's findings. According to the previous case-control study that included 86 women with PCOS between the ages of 18 and 22 years, the prevalence of MetS was 11%, which supported the idea that the prevalence of MetS in women with PCOS was elevated in all age groups (11). However a very resolute finding has been that the prevalence of MetS is dependent upon age and BMI (28, 29). In our study the prevalence of MetS in women with PCOS whose ages ranged from 20-26 years was 12.6%, with a progressive increase to 49.1% in the 27 to 33-yearold group and 65% in the 34 to 40-year-old group. This finding suggested that the prevalence of MetS in our study subjects was highly age-dependent.

In the current study we have observed an approximately two-fold increase in the prevalence of MetS in obese women with PCOS compared with non-obese women. Since most of the women with PCOS (38-88%) are overweight or obese, therefore there is little doubt that adiposity plays an important role in development and maintenance of PCOS and strongly influences the severity of both its clinical and endocrine characteristics in numerous women (30). Obesity appears to be an independent factor for MetS abnormalities; (31, 32) and our results are in accordance with the idea that as BMI increases, the prevalence of high WC and hypertension increases. Nevertheless, fasting serum glucose levels and the rates of dyslipidemia showed no statistically significant difference among the BMI groups, an important finding corroborating the relevance of screening for MetS and other cardiovascular risk factors in all women with PCOS, regardless of obesity. The results of this study have confirmed the high frequency of MetS and its components, in particular a decreased HDL-C level and an increased TG level in women with PCOS. Thus, these women are at increased risk of diabetes mellitus and cardiovascular disease(CVD).Therefore it is important to screen all women with PCOS for cardiovascular risk factors. Recognition and clinical management of this high-risk group are important to prevent CVD and associated mortality in this population.

The current study had some limitations: the relatively small sample size and use of only one diagnostic criterion for MetS (NCEPATPIII definition). In addition, the results might be influenced by the manner in which PCOS and MetS were diagnosed. The lack of a standard definition for MetS in women with PCOS is unfortunate and makes comparison with other studies difficult. We have only included cross-sectional measurements; prospective data from this study will be available in the future to conclusively determine an association between PCOS and CVD in Iranian women.

### Conclusion

In the present study, although 39.5% of all participants had MetS, the majority of those without MetS had at least one criterion by the time of recruitment into this study. Despite the fact that these participants were not diagnosed with MetS by that time, these participants already were at risk for CVD. Therefore in the absence of intervention, they might develop MetS in the future, as the authors found that the frequency of MetS in this population increased with age. The lower frequency of MetS in the younger population and the increasing frequency of this syndrome with increasing age and BMI would be valuable information for the development and implementation of effective management strategies for Iranian women with PCOS.
